# Glutamatergic input–output properties of thalamic astrocytes

**DOI:** 10.1016/j.neuroscience.2011.12.049

**Published:** 2012-03-15

**Authors:** T.M. Pirttimaki, H.R. Parri

**Affiliations:** School of Life and Health Sciences, Aston University, Birmingham, B4 7ET, UK

**Keywords:** astrocytes, glutamate, SIC, gliotransmission, somatosensory, tripartite synapse, CT, corticothalamic, GT, gliotransmission, Lem, Lemniscal, mGluR, metabotropic glutamate receptor, NMDA-R, *N*-methyl-d-aspartate receptor, PSC, post synaptic current, SIC, slow inward current, SSP, spindle stimulation pattern, TC, thalamocortical, VB, ventrobasal, [Ca2^+^]_i_, intracellular calcium

## Abstract

Astrocytes in the somatosensory ventrobasal (VB) thalamus of rats respond to glutamatergic synaptic input with metabotropic glutamate receptor (mGluR) mediated intracellular calcium ([Ca^2+^]_i_) elevations. Astrocytes in the VB thalamus also release the gliotransmitter (GT) glutamate in a Ca^2+^-dependent manner. The tripartite synapse hypothesis posits that astrocytic [Ca^2+^]_i_ elevations resulting from synaptic input releases gliotransmitters that then feedback to modify the synapse. Understanding the dynamics of this process and the conditions under which it occurs are therefore important steps in elucidating the potential roles and impact of GT release in particular brain activities. In this study, we investigated the relationship between VB thalamus afferent synaptic input and astrocytic glutamate release by recording *N*-methyl-d-aspartate (NMDA) receptor-mediated slow inward currents (SICs) elicited in neighboring neurons. We found that Lemniscal or cortical afferent stimulation, which can elicit astrocytic [Ca^2+^]_i_ elevations, do not typically result in the generation of SICs in thalamocortical (TC) neurons. Rather, we find that the spontaneous emergence of SICs is largely resistant to acute afferent input. The frequency of SICs, however, is correlated to long-lasting afferent activity. In contrast to short-term stimulus-evoked GT release effects reported in other brain areas, astrocytes in the VB thalamus do not express a straightforward input–output relationship for SIC generation but exhibit integrative characteristics.

## Highlights

▶VB thalamus afferents were stimulated at a range of frequencies and durations to investigate the interaction of afferent input and astrocytic glutamate output. ▶Acute afferent activity does not result in the evoking of astrocyte glutamate-mediated slow inward currents. ▶Prolonged afferent activity, however, increases SIC frequency in a time-dependent manner. ▶VB thalamus astrocytes therefore exhibit integrative properties that modulate the rate of spontaneous glutamate release.

Glial cells are now considered as active participants in nervous system function (Volterra and Meldolesi, 2005), and the concept of the “tripartite synapse” (Araque et al., 1999) has been advanced to describe the situation where synaptically associated astrocytes act as integral modulatory elements. Astrocytes respond to released neurotransmitters with intracellular calcium [Ca^2+^]_i_ elevations (Cornell-Bell et al., 1990; Porter and McCarthy, 1996; Araque et al., 2002; D'Ascenzo et al., 2007). In turn, astrocytic [Ca^2+^]_i_ elevations can induce the release of gliotransmitters such as glutamate (Pasti et al., 1997; Kang et al., 1998; Parri et al., 2001; Fellin et al., 2004; Perea and Araque, 2005a), ATP (Pascual et al., 2005; Serrano et al., 2006), and d-serine (Henneberger et al., 2010).

In the ventrobasal (VB) thalamus, astrocytes can display spontaneous calcium oscillations *in vitro*, which consequently lead to excitatory *N*-methyl-d-aspartate (NMDA) receptor-mediated currents in thalamic neurons (Parri et al., 2001). These slow inward currents (SICs) are seen in many brain areas and can occur spontaneously or can be evoked by various methods that induce astrocytic Ca^2+^ increases (Parri et al., 2001; Angulo et al., 2004; Fellin et al., 2004; Perea and Araque, 2005a; Kozlov et al., 2006; D'Ascenzo et al., 2007; Navarrete and Araque, 2008; Shigetomi et al., 2008).

However, despite the known ability of synaptic stimulation to evoke astrocytic [Ca^2+^]_i_ elevations and subsequent glutamate release, little is known about the possible physiological roles of SICs and their potential to interact with afferent input in the somatosensory system. To understand these potential roles of astrocytic gliotransmission (GT) release in thalamic function, it is necessary to determine its release properties in relation to afferent activity. We recently found that spontaneous SIC frequency was increased following a period of sustained (>30 min) afferent activity (Pirttimaki et al., 2011). In this study, we sought to determine the dynamic input–output properties of VB thalamus astrocytes by stimulating afferent inputs, which induce metabotropic glutamate receptor (mGluR) mediated [Ca^2+^]_i_ elevations (Parri et al., 2010), and recording astrocytic output in the form of SICs in thalamocortical (TC) neurons.

We found that SIC emergence was unaffected by acute synaptic stimulation but was correlated to the duration of long-term afferent stimulation. VB thalamus astrocytes do not therefore release glutamate in a dynamic way in response to afferent activity but display integrative properties that induce long-lasting changes to astrocyte-neuron signaling.

## Experimental procedures

### Slice preparation

Horizontal slices of VB thalamus were prepared as described previously (Parri et al., 2001) from 12–23-day-old male Wistar rats. All procedures were in accordance with UK Home Office legislation: Animals (Scientific procedures) Act 1986. After removal, the brain was placed in ice cold modified artificial cerebrospinal fluid (aCSF) of composition (mM) NaCl 126, NaHCO_3_ 26, KCl 1, KH_2_PO_4_ 1.25, MgSO_4_ 5, CaCl_2_ 1, glucose 10, pyruvate 5, ascorbic acid 0.3, and indomethacin 0.45. Slices were then maintained at room temperature (23–25 °C) in this solution for a recovery period of 1 h before experimental use.

### Solutions

The standard recording aCSF used in this study was (in mM): NaCl 126, NaHCO_3_ 26, KCl 2.5, KH_2_PO_4_ 1.25, MgSO_4_ 1, CaCl_2_ 2, and glucose 10, unless otherwise stated. As we (Parri et al., 2001) and others have done previously in attempting to enhance NMDA-R mediated current detection, whole cell voltage clamp recordings were conducted in 0-Mg^2+^ (at room temperature), unless otherwise stated. Slices were perfused with the 0-Mg^2+^ solution in the recording chamber. Pharmacological compounds were included in the aCSF as stated in text. Chemicals were obtained from Sigma-Aldrich (St. Louis, MO, USA), unless otherwise stated. Tetrodotoxin (TTX) was obtained from Ascent Scientific (Weston-super-Mare, UK). Fura-2 A.M., Fluo-4 A.M., Alexa-hydrazide 564, and Pluronic F-127 were obtained from Invitrogen (Carlsbad, CA, USA).

### Electrophysiology

The recording chamber and manipulators were mounted on a moveable top plate platform (MP MTP-01, Scientifica, UK). Patch clamp recordings were made using pipettes (2–4 MΩ) containing an internal solution of composition (in mM): KMeSO_4_ 120, HEPES 10, EGTA 0.1, Na_2_ATP 4, GTP 0.5. Currents were recorded using a Multiclamp 700B amplifier, digitized with a Digidata 1440A, and acquired and analysed using pCLAMP (Molecular Devices, CA, USA). Voltage clamp recordings were made at −60 mV, and recordings in which there was a ≥20% change in access resistance during the experiment were excluded from analysis. SICs were analysed using the Event Detection protocols in the Clampfit routine of pCLAMP. Events were accepted as SICs if their amplitude was >20 pA and their time to peak was >20 ms. Data were exported to Sigmaplot (Jandel) for further analysis and plotting.

### Synaptic stimulation

Synaptic stimulation was achieved with a computer-controlled constant current isolated stimulator (STG1002, Multichannel Systems, Germany) and bipolar electrodes, which were placed typically >200 μm from the recorded neurons. Sensory stimulation was achieved by placing a bipolar electrode on the medial lemniscus (Lem), and corticothalamic (CT) afferents were stimulated by a bipolar electrode on the internal capsule. Stimulation protocols were written in the STG1002 interface software. “Protocols” were composed of sequences of “Episodes” of stimulation. After a 5–10 min baseline recording, a stimulation protocol was delivered to one pathway at a time or to both simultaneously. Stimulus episodes were separated by approximately 60-s inter-stimulus intervals. Different stimulus amplitudes ranging from 0.1 mA to 3 mA were tested using trains of 2 ms pulses for 1 s at 50 Hz in randomized order. Different stimulus durations ranging between 2 ms and 10 s were tested at 50 Hz by changing the number of pulses within the train (using sub-maximal stimulus amplitude determined from the evoked EPSC, I_75_). Different frequencies were tested by generating trains of pulses at frequencies between 1 Hz and 500 Hz using constant number of stimuli with I_75_ stimulus amplitudes. A single “spindle stimulation pattern” (SSP) consisted of 22 spikes in duration of 728 ms (Rosanova and Ulrich, 2005). Mean spike rate was 30 Hz, grouped to initial 10 Hz bursts followed by tail of decreasing tonic frequency. In some experiments the SSP was repeated 30 times every 0.6 Hz to mimic the grouping of spindles by the slow (0.6–0.8 Hz) oscillation. Sustained stimulation protocol (10–20 stimuli at 50 Hz every 10 s) as previously described (Pirttimaki et al., 2011) was applied for 30–120 min.

### Fluorescence imaging

**Fig. 1:**
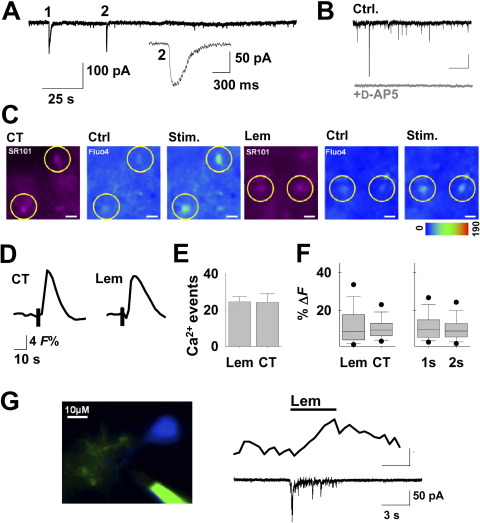
Thalamic afferent stimulation activates astrocytes and neurons. (A) Recording from a TC neuron in presence of TTX (1 μM) showing two spontaneous slow inward currents (SICs). SIC 2 is shown expanded. (B) Representative trace showing spontaneous SIC (black, top) sensitivity to d-AP5 (50 μM) (grey, below). (C) First three panels to the left show a slice area loaded with SR101 (magenta images on the left) and Fluo-4 A.M. Circled SR101-positive astrocytes exhibit Ca^2+^ elevations to CT input (pseudocolor images to the right). Next three panels to the right display a similar experiment to Lemniscal afferent stimulation. (Scale bars: 10 μm). (D) Traces showing averages of 16 CT- and 33 Lemniscal-evoked [Ca^2+^]_i_ responses to the afferent stimulations. (E) Bar graph showing the mean number of responsive astrocytes within the image field of 444 μm×341 μm (Lem, *n*=4 slices; CT, *n*=8). (F) Box plot showing the scatter of relative intensity changes for Lemniscal (*n*=99 [Ca^2+^]_i_ responses) and CT (*n*=194) stimulation, and for 1 (*n*=149) and 2 s (*n*=144) train durations. (G) Panel on the left shows confocal image of Alexa 564-filled TC neuron (blue) and astrocyte (green). To the right: recording from the filled cells in showing that 5-s synaptic stimulation of Lemniscal pathway (top bar) elicits [Ca^2+^]_i_ elevations (middle) in astrocytic processes simultaneously to neuronal EPSC (bottom). For interpretation of the references to color in this figure legend, the reader is referred to the Web version of this article.

After a recovery period of 1 h, slices were loaded with Fluo4 A.M. (Molecular Probes, Eugene, OR, USA) or Fura-2 A.M. by incubating for 40–60 min at 30 °C with 10 μM of the indicator dye and 0.01% pluronic acid. Under these conditions, astrocytes are preferentially loaded (Parri et al., 2001). For astrocytic identification, slices were also loaded with 1 μM Sulforhodamine 101 (SR101), according to *in vitro* methods of Kafitz et al. (Kafitz et al., 2008). Approximately 40 astrocytes (39.7±2.39, *n*=4 slices) could be identified in focus in the VB slice, though this is likely a lower estimate of the number in the visible slice, for example, due to SR101 loading variation. All imaging experiments were performed in aCSF containing Mg^2+^ (1 mM) to record astrocytic responses in physiological conditions. Experiments on imaging with Fura-2 A.M. in Mg^2+^-containing and Mg^2+^-free conditions showed that astrocytic Ca^2+^ elevation responses for the same cells were greater in Mg^2+^-free (0.077±0.005, 340/380 ratio change) than in Mg^2+^-containing aCSF (0.05±0.004 ratio change, *n*=3 slices, 55 cells, *P*<0.01). In patch-clamp experiments designed to maximize SIC detection with Mg^2+^-free aCSF, it would therefore be expected that an increased astrocyte response would also result in a greater probability of detecting any afferent–astrocyte–SIC relationship. Combined patch-clamp and imaging experiments (Fig. 1G) were conducted in Mg^2+^-free aCSF.

The imaged field size was 444 μm×341 μm. The recording chamber and manipulators were mounted on a motorized moveable bridge (Luigs and Neumann, Germany). Fluorescent dyes were excited using an Optoscan monochromator system (Cairn, UK), fitted to a Nikon FN1 upright microscope. Images were acquired for a duration of 0.05–0.1 s every 1–5 s using an Orca-ER CCD camera (Hamamatsu). The short stimulation protocol consisted of trains of 2 ms pulses for 1–2 s at 50 Hz delivered to Lem or CT. Responses were recorded for at least 1 min post stimulation. Acquisition was controlled by Simple PCI software (Hamamatsu).

### Statistics

All quantitative data in the text and figures are presented as mean±SEM. Significance was calculated using unpaired or paired Student's *t*-test as appropriate. Linear correlations (*r*^2^) were tested using Pearson Rank correlation. Statistical significance in the figures is indicated as: * *P*<0.05, ** *P*<0.01, or *** *P*<0.005.

## Results

### VB thalamus astrocytes and neurons respond to synaptic stimulation

Patch clamp recordings from VB thalamus TC neurons revealed spontaneous SICs at low frequencies (∼0.001 Hz), which have been previously shown to be [Ca^2+^]_i_ dependent and TTX-insensitive (Fig. 1A) (Parri et al., 2001; Pirttimaki et al., 2011). The presence of d-AP5 (50 μM) abolished SICs (Ctrl: 16 SICs; d-AP5: 0 SICs; *n*=6 neurons; paired Student's *t*-test *P*=0.003) (Fig. 1B).

Stimulations of Lemniscal and CT inputs at 50 Hz of 1–2 s duration elicited Ca^2+^ elevations which could be detected at the astrocyte soma (Fig. 1C, D). On average 24.5±2.87 astrocytes responded to Lemniscal afferent stimulation (*n*=4 slices) and 24.25±4.65 (*n*=8) to CT stimulation within the imaged area of 444 μm×341 μm (Fig. 1E). The relative intensity change of the Ca^2+^ fluorescence was not different between Lemniscal and CT stimulation (Lem 12.04±0.97ΔF%; CT 10.7±0.4ΔF%; *n*=99, 194 responses, respectively; Student's *t*-test *P*=0.14) nor between 1-s and 2-s long stimulation (1 s 11.53±0.58ΔF%; 2 s 10.8±0.6ΔF%; *n*=149, 144, respectively; *P*=0.4; Fig. 1F), indicating that a maximal cellular [Ca^2+^]_i_ elevation was already induced by the shorter stimulation. Co-loading of SR101 confirmed the astrocytic identity of the responsive cells (Fig. 1C).

Paired recordings from neighboring astrocytes and neurons showed that Lemniscal afferent activation elicited astrocytic [Ca^2+^]_i_ elevations and neuronal EPSCs (*n*=5) (Fig. 1G), indicating that astrocytes and neurons in the same area respond to similar afferent inputs.

### SIC frequency is not increased by acute afferent stimulation

Studies in different brain areas have shown that astrocytic Ca^2+^ responses to afferent stimulation are dependent on parameters such as stimulation intensity, frequency, or duration (Porter and McCarthy, 1996; Pasti et al., 1997; Perea and Araque, 2005b; Beierlein and Regehr, 2006; Parri et al., 2010). The response of astrocytes may not necessarily be linear, indeed, in the somatosensory system, whisker stimulation at 5 Hz was shown to be the most effective at evoking Ca^2+^ responses in the barrel cortex *in vivo*, with 1 Hz and 10 Hz being least effective (Wang et al., 2006). Although many studies have demonstrated the Ca^2+^ dependence of GT release (Araque et al., 2000; Bezzi et al., 2004; Liu et al., 2011), there is continuing debate on the precise Ca^2+^ signal necessary for such release (Fiacco et al., 2007; Shigetomi et al., 2008; Hamilton and Attwell, 2010), which may be complicated by the difficulty in identifying the relevant astrocytic compartment in which a particular Ca^2+^ elevation is necessary for GT release. At present, therefore, predicting GT release by monitoring astrocytic Ca^2+^ activity does not seem a reliable method. To understand the contribution of astrocytic GT release to brain function, however, we must understand the conditions under which astrocytes release GTs, and for a sensory system: in response to which particular activity.

Therefore, to directly measure the relevant physiological output of the astrocytic GT to neuron signaling, we recorded SICs from TC neurons and tested a variety of afferent activity patterns of different frequencies and duration to Lemniscal and corticothalamic afferents. Spontaneous SICs were observed in 40 of 76 (52.6%) recordings with a mean frequency of 0.16±0.03 SICs/min over a 5–10 min control period. The frequency of SICs (expressed as SICs/min) after each stimulus episode was calculated and compared with this pooled spontaneous frequency.

**Fig. 2:**
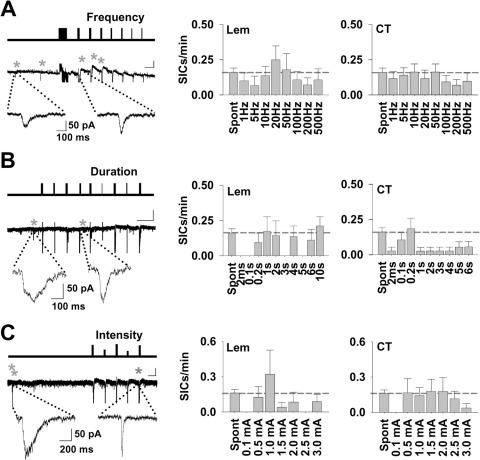
Synaptic stimulation does not evoke SICs. (A) Line diagram (top) illustrates the protocol of the pattern of episodes of 50 stimuli delivered at different frequencies (1–500 Hz) to CT input. The responses of the TC neuron are shown in the trace below, with SICs indicated by asterisks (Scale bar: 50 s, 50 pA). Expanded example SICs are shown beneath. (Right) Bar graphs show the relationship of mean frequency of SICs (SICs/min) to different stimulus frequencies for the Lemniscal (*n*=21 neurons) and CT (*n*=30) inputs. (B) Line diagram (top) illustrates the protocol of the pattern of stimulation delivered at different duration (2 ms–6 s) to CT input. The responses of the TC neuron are shown in the example trace below (Scale bar: 60 s, 50 pA), with SICs indicated by asterisks and examples are expanded beneath. (Right) Bar graphs summarizing the mean frequencies (Lem, *n*=23; CT, *n*=40). (C) Line diagram (top) illustrates the protocol of the pattern of stimulation delivered at different intensities (0.5–3 mA) to CT input. The responses of the TC neuron are shown in the example trace below, with SICs indicated by asterisks (Scale bar: 25 s, 50 pA). Expanded example SICs are shown beneath. (Right) Bar graphs show the relationship of mean frequency of SICs to different stimulus intensities (Lem, *n*=25; CT, *n*=28). Dashed line in bar graphs indicates mean spontaneous SIC frequency. The neuronal post synaptic currents and stimulation artefacts (vertical lines) are truncated for clarity in (A–C).

To determine the afferent frequency dependence of SIC frequency, a protocol consisting of trains of 50 stimuli (range 1–500 Hz) was applied to Lemniscal and CT afferents. The order of the frequency trains were changed between experiments (Fig. 2A). For Lemniscal input 61% of TC neurons expressed SICs during the frequency stimulation protocol (*n*=21 neurons), whereas 53% of neurons (*n*=30) expressed SICs with CT input. SIC frequency was not significantly increased by varying the frequency for either Lemniscal (Student's *t*-test for each stimuli *P*>0.1) or CT input (*P*>0.6) (Fig. 2A).

To determine the afferent stimulus duration dependence of SIC frequency, a protocol consisting of trains of 2 ms stimuli at 50 Hz (range 2 ms–10 s) was applied (Fig. 2B). Of 23 neurons, 39% expressed SICs during Lemniscal stimulation, whereas 27% SICs occurred during CT stimulation (*n*=40). The frequency of apparent evoked SICs for any particular stimulus was not increased compared with spontaneous, while frequency following some trains was significantly lower (Lem: 2 ms, 0.1 s, 3 s, 5 s, Student's *t*-test *P*<0.05; CT: 2 ms, 1–6 s, *P*<0.05).

Experiments in Fig. 2A, B were conducted using a submaximal (75%) stimulus intensity. This was determined using a protocol consisting of 50 stimuli at 50 Hz (1 s) at constant current intensities of 0.1–3 mA. Analyzing the data for SIC frequency (Fig. 2C) showed that SICs followed Lemniscal and CT stimulation in 28% and 44% of the cells tested (*n*=25, 28, respectively), but frequency was not increased compared with spontaneous (Lem: 1–1.5 mA and 2.5 mA less than control, *P*<0.03, rest *P*>0.2; CT: 0.1 mA and 3 mA less than control, *P*=0.02; rest *P*>0.2). In addition, there was no correlation between increasing stimulus frequency, stimulus train duration, nor stimulus magnitude and SIC frequency (Pearson's correlation, *r*^2^<0.2, *P*>0.06 for all groups).

### SIC timing is independent of synaptic stimulation

**Fig. 3:**
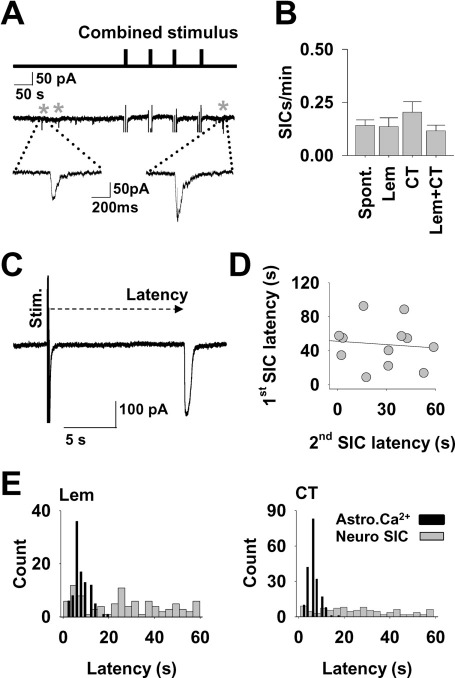
SIC timing is independent of synaptic stimulation. (A) Line diagram (top) with vertical bars representing simultaneous Lemniscal and CT stimulus episodes. Below is an example trace showing corresponding synaptic responses (vertical lines represent stimulus artefacts and inward currents, the neuronal post synaptic current is truncated for clarity) and SICs (marked with asterisks). (B) Bar graph summarizing the SIC frequency during different stimulus protocols (*n*=27 neurons). (C) Example of a 0.2-s-long stimulus at 50 Hz delivered to the CT afferent with a SIC following within a few seconds, illustrating the measurement of SIC latency. (D) Scatter plot showing the latency of SICs following a stimulus against the latency of a second SIC to a subsequent stimulus (*r*^2^=0.008). (E) Frequency histograms showing the distribution of SIC latencies (grey bars) and Ca^2+^ delays (black bars) (*n*=12 slices) following Lemniscal (left) (*n*=96 neurons) and CT stimulation (right) (*n*=125 neurons).

It has been proposed that astrocytes function as coincidence detectors in the hippocampus, since Ca^2+^ responses were modulated during simultaneous Schaffer collateral and alveus stimulation, particularly at theta frequencies (Perea and Araque, 2005a). We tested the hypothesis that such thalamic coincidence detection would lead to increased SICs by simultaneously stimulating Lemniscal and CT inputs. Stimulus parameters that were most often followed by SICs in Fig. 2 were selected (Lem: 10 s stimuli at 20 Hz, CT: 0.2 s at 50 Hz). Each combination was repeated four times, first separately and then simultaneously (Fig. 3A). Although 85% of the TC neurons (*n*=27) expressed SICs during the stimulation protocols, mean SIC frequency was not significantly increased either with single pathway stimulation or simultaneous stimulation (Spont. 0.142±0.026 SICs/min; Lem 0.136±0.041 SICs/min, Student's *t*-test *P*=0.9; CT 0.204±0.049 SICs/min, *P*=0.2; and simultaneous Lem/CT 0.116±0.027 SICs/min, *P*=0.6; Fig. 3B). The data support the finding that SIC frequency is not increased by acute synaptic stimulation (Fig. 2) and also indicates that VB thalamus astrocytes do not function as coincidence detectors for the release of glutamate.

If SICs are elicited by synaptically induced astrocytic [Ca^2+^]_i_ elevations in a deterministic manner, then the prediction is that repeated stimulation will result in repeated SICs, and that there will be a consistent delay between stimulus and SIC emergence in the same tripartite synapse. This was found in hippocampus, where following repeated application of a stimulus there was a linear correlation between the delays of the first and second observed SIC (Fellin et al., 2004). We tested this hypothesis in the VB thalamus, and measured the latencies from recorded SICs to the preceding stimulus (Fig. 3C), and in calcium-imaging experiments from the stimulus to peak [Ca^2+^]_i_ elevation. No correlation was detected between the latencies of first and second SICs to repeated stimulus (*r*^2^=0.008, Pearson's correlation *P*=0.8; Fig. 3D). Also, while histograms of the latencies of astrocytic [Ca^2+^]_i_ elevations displayed characteristic peaks at around 7 s (Fig. 3E) (Lem 7.86±0.3 s; CT 6.39±0.18 s; *n*=12 slices, 99, 194 astrocytes, respectively; Student's *t*-test *P*=0.00004), this was not the case for SICs, with latencies of SICs following Lemniscal stimulation ranging from 0.37 to 173.3 s, those following CT stimulation from 0.85 and 100.7 s (Fig. 3E). Plotted latency histograms do not indicate a discernable peak for either afferent input. These results support a situation where synaptic stimulation induces astrocytic [Ca^2+^]_i_ elevations but not SICs.

### SIC frequency is not affected by afferent neurotransmitter release

**Fig. 4:**
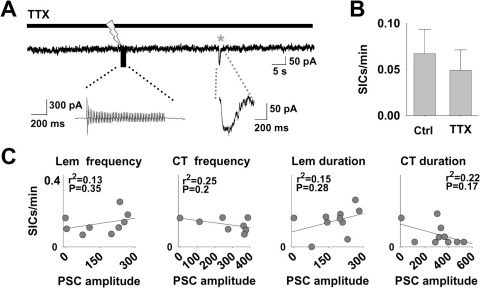
SIC frequency is not correlated to post-synaptic glutamate effect. (A) Trace of a recording following the addition of TTX which blocks elicited PSC to afferent stimulation (lightning bolt) and only results in a stimulus artefact (expanded below). A spontaneous SIC (grey asterisk) occurs during the recording (expanded below). (B) Mean SIC frequency during control stimulation and following the addition of TTX (*n*=10 neurons). (C) Mean SIC frequency plotted against the mean PSC amplitude for the different stimulus protocols (CT analysis: *n*=125 neurons, Lem: *n*=96 neurons).

A dependence of SIC emergence on afferent synaptic input is reliant on the action of the released neurotransmitter on the astrocytes. Previous reports have indicated that SIC generation is dependent on synaptic activity by the sensitivity of SIC emergence to TTX and Cd^2+^, which inhibit neurotransmitter release from synaptic terminals (Perea and Araque, 2005a; D'Ascenzo et al., 2007). Following a control stimulation protocol, we bath applied TTX (1 μM), and the protocol was repeated (Fig. 4A). Synaptic inward currents were completely blocked by TTX (Ctrl 570.5±49.3 pA; TTX 0 pA; paired Student's *t*-test, *P*=0.00008; *n*=10; Fig. 4A). However, SIC frequency was unaffected (Ctrl 0.067±0.03 SICs/min; TTX 0.05±0.02 SICs/min; paired Student's *t*-test, *P*=0.5; *n*=10; Fig. 4A, B).

It is possible that GT release and SIC generation is dependent on the amount of glutamate released by VB thalamus afferent inputs that activates astrocytic receptors. Synaptic input to a VB thalamus slice activates neighboring neurons and astrocytes (Fig. 1), we therefore used the neuronal postsynaptic current (PSC) amplitude as a measure of neurotransmitter glutamate released upon synaptic activation and compared this with SIC frequency in the same neuron. There was no correlation between PSC amplitude and SIC frequency for any of the previously described (Fig. 2) protocols (Fig. 4C).

### Physiological state-dependent stimulation does not increase SIC frequency

The thalamus exhibits different activity depending on conscious state, which can be modulated by brainstem afferents, for example, sleep is characterized by hyperpolarized TC neurons exhibiting delta oscillation, whereas in awake state, cholinergic and noradrenergic afferents from the brain stem depolarize TC neurons into relay or “tonic” mode (McCormick, 1992; Castro-Alamancos, 2002). Therefore, there was the possibility that these afferents were also “gating” astrocytic responsiveness in the same way that they gate TC neuron responsiveness. To determine this, we recorded astrocytic [Ca^2+^]_i_ elevations and neuronal SICs following afferent activation in the presence of cholinergic, adrenergic, or both agonists.

Stimulation protocols were applied in control aCSF and then following the bath application of the muscarinic agonist carbachol (50 μM) and the β adrenergic agonist isoproterenol (50 μM).

**Fig. 5:**
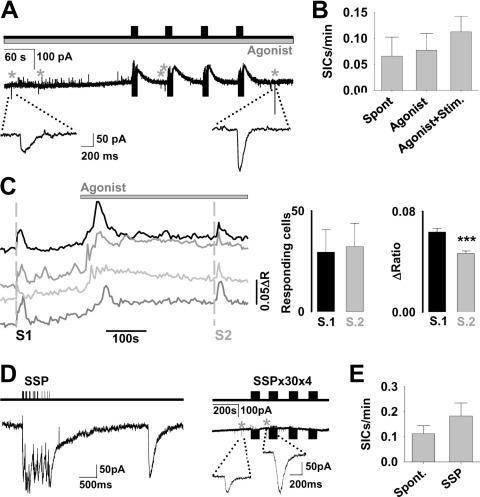
Physiological modulators or state-dependent stimulation do not increase SIC frequency. (A) Line diagram (top) illustrating the pattern and timing of episodes of 50 stimuli delivered to CT and Lem afferents in the presence of the exogenous agonists carbachol (50 μM) and isoproterenol (50 μM). The responses of the TC neuron are shown in the example trace below, with SICs indicated by asterisks. Expanded example SICs are shown beneath. (B) Bar graph summarizing the SIC frequency for different conditions (Spont, *n*=12; Agonist, *n*=16 recordings). (C) Ratio traces from four example astrocytes in an imaged slice showing responses to a 2 s 50 Hz combined afferent input in control conditions (S1) and following exposure to isoproterenol/carbachol (S2) (*n*=119 astrocytes, 4 slices). Bar graphs to the right illustrate the number of responding astrocytes and magnitude of ratio change to the stimuli in the two conditions. (D) Leftmost: line diagram (top) illustrating the SSP stimulation pattern. Trace below shows the post synaptic current in response to SSP stimulation (left) followed with a delayed SIC (right). To the right: line diagram (top) illustrating the 4 repeated blocks of 30 SSP patterns with recorded current trace below. SICs are asterisked and expanded beneath. (E) Bar graph showing the mean SIC frequency before and during the SSP protocols (*n*=23 recordings). The neuronal post synaptic current and stimulation artefacts (vertical lines) are truncated for clarity in (C) and (E) (right).

Because of the similar physiological effects and close agreement of carbachol and isoproterenol, data from agonist application experiments (carbachol, isoproterenol, carbachol+isoproterenol) is illustrated pooled as “agonist.” Application of agonists did not increase spontaneous SIC frequency, nor increase SIC frequency following afferent stimulation (Ctrl spont. 0.066±0.037 SICs/min vs. spontaneous with agonist wash-on 0.077±0.03 SICs/min, Student's *t*-test *P*=0.8, *n*=12, 16, respectively; Ctrl spont. vs. agonists with stimulation 0.099±0.046, *P*=0.6, *n*=12, 16, respectively; Fig. 5A, B).

An action of the agonists on VB thalamus astrocytes was verified by conducting [Ca^2+^]_i_-imaging experiments using the ratiometric indicator Fura-2. Carbachol and isoproterenol co-application elicited an increase in astrocytic [Ca^2+^]_i_ (Fig. 5C) (Peak increase 0.059±0.003, *n*=119 astrocytes, 4 slices). Analysis of [Ca^2+^]_i_ responses to synaptic stimulation revealed that the number of responding astrocytes was not affected (Control: 29.5±11.01, agonist: 32.25±11.4, *n*=4 slices) but that [Ca^2+^]_i_ elevations to afferent input were slightly reduced in the presence of agonist (Control ratio change 0.063±0.003, agonist ratio change 0.046±0.002, *n*=119 astrocytes, 4 slices, *P*<0.001). VB thalamus astrocytes, therefore, respond to the applied agonists, but this does not increase the likelihood of SIC generation to afferent stimulation.

During sleep, the cortex and thalamus are engaged in low-frequency oscillations (Castro-Alamancos, 2004; Steriade, 2006). In particular, sleep spindles, an intermittent frequency pattern which have been shown to induce synaptic plasticity in the thalamocortical network (Rosanova and Ulrich, 2005), increase in frequency. We therefore stimulated CT afferents with protocols mimicking sleep spindles and slow oscillations as described by Rosanova and Ulrich (Rosanova and Ulrich, 2005). Spindle stimulation pattern (SSP) stimulation was delivered via CT afferents either as a single SSP stimulation (Fig. 5D, E), or a train of 30 SSPs repeated four times (SSP×30×4; Fig. 5D, E). SSP stimulation did not increase SIC frequency significantly (Ctrl 0.21±0.098 SICs/min; SSP 0.39±0.14 SICs/min; paired Student's *t*-test *P*=0.4; *n*=6 neurons). Nor did trains of SSP; 47% of neurons showed SICs during the SSP×30×4 protocol with an average SIC frequency of 0.076±0.024 SICs/min compared with spontaneous 0.109±0.039 SICs/min (Paired Student's *t*-test *P*=0.53; *n*=17).

Interventions to investigate possible neuromodulator or synaptic input state dependent effects, therefore, had no effect on SIC frequency.

### Prolonged afferent stimulation is required to increase SIC frequency

**Fig. 6:**
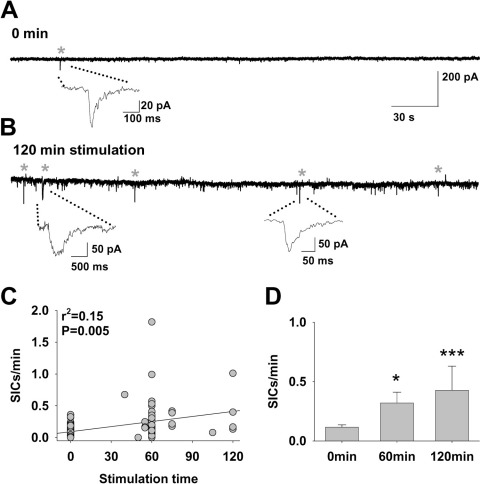
Integration of afferent input increases spontaneous SIC frequency. (A) Trace of a recording from an un-stimulated control slice (SICs indicated with asterisks). (B) Trace of a recording from a TC neuron in a slice following a 120 min of prolonged intermittent stimulation. (C) Scatter plot showing a positive correlation of SIC frequency with intermittent stimulus duration. (D) Mean SIC frequency following different intermittent stimulus durations (0 min, *n*=26; 60 min, *n*=19; 120 min, *n*=4 neurons). Statistical significance is presented as * *P*<0.05, ** *P*<0.01, or *** *P*<0.005.

We recently found that following prolonged synaptic stimulation (50 Hz bursts every 10 s for 60 min) there was a sustained increase in the frequency of spontaneous SICs that lasted for up to an hour without further synaptic input, which we termed long-term enhancement of SICs (Pirttimaki et al., 2011). Having found in this study that short stimulus patterns (Figs. 2–5) do not increase SIC frequency, we investigated the relationship between sustained afferent activity duration and SIC frequency by varying stimulus durations and then recording SIC frequency after ceasing stimulation (Fig. 6A, B). Spontaneous SIC frequency showed a significant correlation with stimulation time (*r*^2^=0.15, *P*=0.005; Fig. 6C). Mean SIC frequency after 60 min and 120 min stimulation were significantly higher compared with un-stimulated coonditions (0 min 0.116±0.02, *n*=26; 60 min 0.32±0.09, *n*=19; *t*-test *P*=0.016; 120 min 0.427±0.2, *n*=4; *P*=0.0015 compared with 0 min; Fig. 6D).

## Discussion

The main finding of this study is that astrocytes in the VB thalamus do not readily generate SICs by release of glutamate in response to short periods of synaptic stimuli, though such stimuli have been shown to cause astrocytic [Ca^2+^]_i_ elevations. A simple tripartite synaptic signaling model whereby afferent input induce elevations, which then release glutamate in a deterministic manner, does not therefore hold true for the VB thalamus. Over long periods, however, astrocytes integrate afferent activity, and the rate of spontaneous glutamate release is increased.

Despite increasing realization of non-housekeeping roles for astrocytes in brain function (Volterra and Meldolesi, 2005; Perea et al., 2009), there is continuing debate concerning the release of GTs, their mechanism of release, [Ca^2+^]_i_ dependence, and physiological impact (Fiacco et al., 2007; Petravicz et al., 2008; Agulhon et al., 2010; Hamilton and Attwell, 2010). A range of GTs is known to be released by astrocytes, for example, ATP, d-serine, GABA, and glutamate, and there is pharmacological, imaging, and selective SNARE-protein mouse knockout evidence for the vesicular release of ATP and glutamate (Bezzi et al., 2004; Perea and Araque, 2007), but the relationship between stimuli such as synaptic activation, resultant [Ca^2+^]_i_ elevations and release is proving more difficult to understand.

### Astrocytic input–output properties in the somatosensory system

Synaptic afferent activity via Lemniscal or corticothalamic inputs induces mGluR-dependent [Ca^2+^]_i_ elevations in VB thalamus astrocytes (Parri et al., 2010), and VB thalamus astrocytes also release glutamate in a [Ca^2+^]_i_-dependent manner (Pirttimaki et al., 2011). Results from somatosensory cortex *in vivo* show that astrocytic [Ca^2+^]_i_ elevations caused by whisking display preferred whisking frequency (Wang et al., 2006) and in visual cortex *in vivo* astrocytes also display orientation selectivity (Schummers et al., 2008). Such activity in response to synaptic input, if translated into GT output, could have profound effects and roles in sensory processing. We investigated this in the VB thalamus by recording SICs in TC neurons as a direct measure of astrocytic glutamate release and its potential physiological impact. However, we did not find that patterns consisting of different frequencies or train duration up to 10 s resulted in more SICs than seen spontaneously. Adding neuromodulators that affect TC neuron relay (McCormick et al., 1993) or stimulating with sleep spindle patterns did not induce SICs either. Our results, however, show that in response to sustained periods of afferent activity, astrocytes increase their rate of spontaneous SICs, and this is correlated to the duration of activity.

Studies *in vivo* in the rat VB thalamus have shown that whisker stimulation releases the GT homocysteic acid (HCA) (Do et al., 2004) via glutamate receptor activation (Benz et al., 2004). HCA is an agonist at NMDA receptors; it might therefore be possible that the GT mediating SICs could be HCA. However, HCA levels increased within a few minutes of whisker stimulation and were also elevated by isoproterenol, in contrast to the pattern seen for SICs. It therefore seems that a complex situation exists in the VB thalamus whereby afferent input results in different GTs acting on NMDA receptors with different kinetics and on different temporal scales.

Our findings in this study extend our recent work showing that following sustained activity, SIC frequency is increased by an mGluR-dependent mechanism. Thus, our results may indicate a dual role for astrocytic mGluR activation, so that short immediate activation elevates astrocytic [Ca^2+^]_i_ (Parri et al., 2010), but does not reliably induce glutamate release; however, repetitive afferent activity, perhaps by recruiting other cellular pathways, integrates the repetitive mGluR activation and up-regulates spontaneous glutamate release.

### Comparison to other brain areas

Our results are in contrast to some reports from the hippocampus (Fellin et al., 2004) and nucleus accumbens (D'Ascenzo et al., 2007), where afferent stimulation has been shown to reproducibly induce SICs. This may indicate that there are regional differences in the brain in the interaction between neurons and glia and the physiological roles of astrocytes. It may therefore be significant that much evidence about the roles of astrocytes in synaptic modulation and plasticity is based on studies in the hippocampus, a part of the brain involved in learning and memory and a model system for investigating synaptic learning, whereas in the VB thalamus, a sensory relay nucleus, synapses transmitting sensory information do not undergo such radical short- and long-term potentiation as the hippocampus.

### Functional implications

Although the functional roles of GT release in the hippocampus in modulating synaptic transmission and plasticity are immediately apparent, the roles of glutamate release in the VB thalamus are less clear. Rather than being directed at the synapse, VB thalamus astrocytic glutamate release seems targeted extrasynaptically, and resultant SICs can cause neuronal firing (Pirttimaki et al., 2011). It may therefore be that astrocytic glutamate provides a thalamic excitatory drive for the generation of specific TC neuron firing patterns. Our experiments specifically recorded glutamate events; however, it is possible that following acute afferent activity other GTs are released such as arginine (Do et al., 1994), GABA (Jimenez-Gonzalez et al., 2011), prostaglandins, or ATP, and that these exhibit different input–output relationships. Indeed, it has already been shown that astrocytic ATP release generates thalamic k-complexes which occur during sleep (Lorincz et al., 2009). A key feature of VB thalamus astrocytic glutamate release that leads to SICs is that the signaling is not immediately evoked by afferent input but that the amount afferent activity over long periods affects the spontaneous emergence of astrocyte-neuron glutamate signaling. The integrated generation of SIC excitatory drive following sustained sensory input suggests a mechanism that would become more influential following a period of awake behaving sensory input. However, the increase in SIC activity can also be produced by corticothalamic input, which is the predominant afferent input during sleep states. Further studies are therefore necessary to determine the precise role of astrocytic glutamate release and SICs in different thalamocortical oscillatory activities associated with specific conscious dependent states.

## Author contribution

All experiments were carried out in the department of Life and Health Sciences at the Aston University. H.R.P. and T.M.P. designed the experiments, T.M.P. and H.R.P. performed experiments, T.M.P. analysed the data and prepared illustrations. Manuscript was written by H.R.P. and T.M.P.
